# New mechanisms of action and signaling by TNF-α

**DOI:** 10.1186/ar3590

**Published:** 2012-02-09

**Authors:** Lionel B Ivashkiv

**Affiliations:** 1 Arthritis and Tissue Degeneration Program, Hospital for Special Surgery, New York, NY 10021, USA; 2 Department of Medicine and Immunology and Microbial Pathogenesis Program, Weill Cornell Medical College, New York, NY 10065, USA

## 

TNF-α is a key pathogenic factor in inflammatory arthritis. Rapid and transient signaling and functional responses of cells to TNF-α, such as activation of NF-γB and MAPKs, are well known. These signaling mechanisms are widely assumed to be functional in cells chronically exposed to TNF-α and to mediate the pathogenic effects of TNF-α in chronic inflammation. We investigated the responses of primary macrophages to TNF-α over the course of several days and compared patterns of signaling and gene expression to RA synovial macrophages. The acute inflammatory response to TNF-α subsided after several hours and was followed by an IFN response characterized by sustained expression of STAT1 and downstream target genes. TNF-α-mediated induction of an IFN response was mediated by IFN-β and was sensitive to inhibition by Jak inhibitors. Concomitantly TNF-α induced a state of macrophage resistance to the homeostatic cytokines IL-10 and IL-27. Microarray analysis demonstrated that sustained TNF-α signaling induced expression of novel genes not appreciated to be 'TNF-inducible', but are highly expressed in RA synovial macrophages. Induction of an IFN response and abrogation of homeostatic cytokine signaling was also observed in RA synovial macrophages and likely contributes to the pathogenic actions of TNF-α during arthritis.

Subsequently and surprisingly, TNF-α induced a tolerant state in macrophages, with diminished cytokine production on lipopolysaccharide (LPS) challenge and protection from LPS-induced lethality. TNF-α-induced cross-tolerization was mediated by coordinate action of two inhibitory mechanisms, suppression of LPS-induced signaling and chromatin remodeling. Mechanistically, TNF-α-induced cross-tolerance was distinguished from TLR-induced tolerance by strong dependence on the nuclear kinase GSK3, which suppressed chromatin accessibility and promoted rapid termination of NF-γB signaling by augmenting negative feedback by A20 and IγBα. These results reveal an unexpected homeostatic function of TNF-α and provide a GSK3-mediated mechanism for preventing prolonged and excessive inflammation. This homeostatic mechanism may be compromised during RA synovitis, possibly by hypomorphic alleles of *TNFAIP3 *(encodes A20) or by cytokines that suppress A20 expression or antagonize its function. These data suggest that augmenting homeostatic functions and signals and thereby rebalancing the pro- *versus *anti-inflammatory profile of TNF-α may represent an efficacious alternative therapeutic approach to suppress chronic inflammation.

Overall, the data reveal novel signals and functions of TNF-α and that are likely operative during chronic inflammation and RA synovitis. Targeted inhibition of these non-traditional functional components of the TNF-α response may be efficacious in alleviating chronic inflammation while preserving acute TNF-α responses and host defense against infections.

